# Role of charge in enhanced nuclear transport and retention of graphene quantum dots

**DOI:** 10.1038/s41598-024-69809-2

**Published:** 2024-08-16

**Authors:** Gorav Gorav, Vrushali Khedekar, Geetha K. Varier, P. Nandakumar

**Affiliations:** Department of Physics, Birla Institute of Technology and Science, Pilani, K. K. Birla Goa Campus, Zuarinagar, Goa 403726 India

**Keywords:** Time-lapse confocal imaging, FG nucleoporins, Nuclear pore complex, Graphene quantum dot, Nuclear transport, Biological fluorescence, Nanoscale biophysics, Permeation and transport

## Abstract

The nuclear pore complexes on the nuclear membrane serve as the exclusive gateway for communication between the nucleus and the cytoplasm, regulating the transport of various molecules, including nucleic acids and proteins. The present work investigates the kinetics of the transport of negatively charged graphene quantum dots through nuclear membranes, focusing on quantifying their transport characteristics. Experiments are carried out in permeabilized HeLa cells using time-lapse confocal fluorescence microscopy. Our findings indicate that negatively charged graphene quantum dots exhibit rapid transport to the nuclei, involving two distinct transport pathways in the translocation process. Complementary experiments on the nuclear import and export of graphene quantum dots validate the bi-directionality of transport, as evidenced by comparable transport rates. The study also shows that the negatively charged graphene quantum dots possess favorable retention properties, underscoring their potential as drug carriers.

## Introduction

The nucleus, a specialized organelle within eukaryotic cells, serves as the repository for the cell’s genetic material, stored in the form of chromatin-a complex comprising DNA and proteins. Its crucial functions encompass the regulation of gene expression, DNA replication, DNA repair, and the oversight of cell cycle progression. The nuclear membrane physically separates the nucleus from the cytoplasm as a dynamic barrier. Nuclear pore complexes (NPCs), situated in the nuclear membrane and consisting of large protein assemblies, facilitate the transport of molecules between the nucleus and the cytoplasm^1–4^. Molecules with a molecular weight of less than 40 kDa (less than 9 nm in diameter) undergo passive diffusion into the nucleus, while larger molecules utilize an active transport mechanism that requires energy^4–8^. Distinct signal molecules, such as the nuclear localization signal (NLS) and nuclear export signal (NES), play a crucial role in enabling the active transport of cargo into and out of the nucleus. Karyopherins (importins and exportins) present in the cells have binding sites for NLS and NES to mediate this transport. The complex formed by the karyopherins and the cargo molecules undergoes translocation through NPCs, facilitated by interactions with phenylalanine-glycine (FG) nucleoporins (Nups)^4,9,10^.

Nuclear translocation is a vital process in all living cells, and comprehending this intricate phenomenon requires insight into the internal structure of NPCs. NPCs exhibit 8-fold rotational symmetry and consist of approximately 30 distinct types of Nups. Human cell NPCs primarily feature Nups 214, 88, and 62 on the cytoplasmic side, Nups 98, 62, 58, and 54 on the central ring, and Nups 153 and 50 on the nuclear side of the nuclear membrane^11^. These Nups contain phenylalanine and glycine amino acid repeats connected through hydrophilic linkers^1,2,12^. The structure of FG Nups can take the form of an extended coil, collapsed coil, or a combination, depending on the amino acid charge content. One-third of FG Nups are intrinsically disordered proteins (IDPs), forming a selective hydrogel mesh along the central axis that is dynamic in nature^13^. During nucleocytoplasmic transport, the flexibility of FG-IDPs is constrained by cargo molecules, resulting in entropy reduction. While small molecules, less than 9 nm, have minimal impact on IDP entropy during NPC diffusion, larger molecules (greater than 9 nm) restrict IDP movements, leading to decreased FG-IDP entropy. The hydrophobic interaction between the cargo complex (cargo+NLS+karyopherins) and IDPs decreases the enthalpy of the system to compensate for the entropy decrease, a phenomenon known as enthalpy-entropy compensation^14,15^. Small biomolecules diffuse through gaps between IDP structures, while hydrophobic FG- IDPs act as a barrier for larger molecules^8,13,14,16^. Previously, the sole recognized interaction assisting in overcoming this barrier was the hydrophobic interaction between the cargo complex and FG Nups. However, recent research from multiple groups has documented that electrostatic interactions between negatively charged cargo and positively charged FG Nups also contribute to nuclear transport^17–19^.

Investigations on biomolecular transport through nuclear membranes have been an important technique for probing nuclear pore complexes. Inert dextran molecules, available in various sizes, are widely employed model systems to explore transport mechanisms through nuclear membranes. The dextran molecules, appropriately labeled with fluorescent dyes like fluorescein isothiocyanate (FITC) or tetramethyl rhodamine isothiocyanate (TRITC), are utilized in experiments to assess their nuclear uptake using confocal fluorescence microscopy. These studies have provided valuable insights into nuclear transport characteristics, including the size of nuclear pores and the permeability properties of the nuclear membrane. The molecular weight of transporting molecules significantly influences their nuclear uptake^5,7,20,21^. Notably, the nuclear accumulation and retention time of dextran within the nucleus exhibit a linear dependence on molecular weight^21^. Additionally, the rate of nuclear transport of dextran is influenced by the excluded volume effect resulting from molecular crowding within the nucleus. Given that human cell nuclei are 30–40 % filled with chromatins^22^, the concentration of dextran increases nuclear crowding, leading to steric repulsion. It can be seen that FITC dextran is excluded from the chromatin region and nucleoli. However, it has been observed that molecular crowding is contingent on the interaction between the cargo and the environment, and cargo with a weak interaction can overcome crowding behavior and steric repulsion^23^.

In recent years, nanoparticles have garnered significant research attention as potential biomolecular probes for nuclear transport studies. Nanoparticles prove effective in targeted drug delivery, mitigating the side effects of chemotherapy^24–29^. They also find application as probes in various microscopic techniques, including two-photon microscopy, confocal microscopy, and fluorescence resonance energy transfer microscopy^29–31^. Due to their substantial surface-to-volume ratio, nanoparticles facilitate the attachment of peptides, aiding in cellular, nuclear, and endosomal membrane escape^24–27,32^. Studies indicate that the cellular and nuclear uptake of nanoparticles depends on their elasticity, size, and surface properties^24–26,29^. While gold nanoparticles have been widely used in these studies, graphene quantum dots (GQDs) have emerged as another potential probe of interest, attributed to their low toxicity, biocompatibility, and oxygen-rich functional group. GQDs are also advantageous for microscopic imaging due to their autofluorescence properties, high quantum yield, photo-stability, and reduced photo-bleaching^30,31,33,34^.

In the present study, we investigate the kinetics of nuclear import and export of GQDs using time-lapse confocal fluorescence microscopy. Experiments are conducted in digitonin-permeabilized cell lines, a well-established system for studying transport under transient conditions. Digitonin selectively permeabilizes the cell membrane while keeping the nuclear membrane intact, enabling the direct study of molecular transport through the nuclear membrane. GQDs, employed as potential cargo molecules in our studies, carry a carboxyl group and are negatively charged. While different groups have observed the cellular and nuclear uptake of GQDs, the kinetics of their transport and translocation rates remain understudied and unquantified. Our findings reveal the rate constants of GQD transport through the HeLa cell nuclear membrane, shedding light on various aspects of nuclear import and export of GQDs.

## Materials and methods

### Materials

The following chemicals used in the experiments are procured from Himedia Laboratories Private Limited, India: Dulbecco’s Modified Eagle Medium (DMEM), Fetal Bovine Serum (FBS), Trypsin-EDTA, Dulbecco’s Phosphate Buffer Saline (PBS), Antibiotic Antimycotic Solution, N-(2-Hydroxyethyl)-piperazine ethane sulfonic acid (HEPES), Potassium Acetate (KAc), Sodium Acetate (NaAc), Magnesium Acetate (MgAc), Ethylene glycol-bis(2-aminoethyl ether)-N,N,N’,N’-tetra acetic acid (EGTA), 1,4 Dithiothreitol (DTT), Protease Inhibitor, and Digitonin. Additionally, Rabbit Reticulocyte Lysate (RRL) is obtained from Promega, USA. The GQDs utilized in the study are prepared by S.D. Hiremath as per the procedure in Bhosle et al.^35^, with a final stock concentration of 2 mg/ml. The GQDs used in nuclear transport studies are negatively charged with a zeta potential value of $-32.4$ mV. The size of GQDs characterized using transmission electron microscopy is approximately 6 nm. The hydrodynamic size observed through the particle size analyzer is 10.4 nm. The characterization data are available in Supplementary Information (Fig. S1). All reagents used in the study are of analytical grade.

A stock solution of digitonin is prepared by dissolving 40 mg of digitonin powder in 1 ml of dimethyl sulfoxide^36^. This stock solution is further diluted with the complete transport buffer, and a 40 $\upmu$g/ml digitonin solution is employed for permeabilization. The transport buffer comprises 20 mM HEPES, 110 mM KAc, 5 mM NaAc, 2 mM MgAc, and 0.5 mM EGTA, adjusted to pH 7.3. To make a complete transport buffer, 2 mM DTT and 1 $\upmu$g/ml of Protease Inhibitor cocktail (aprotinin, leupeptin, and pepstatin) are added to the transport buffer while maintaining the same pH^37^.

For import experiments, two types of import mixtures are employed: one involving the complete transport buffer containing RRL (prepared by combining 12.5 $\upmu$l dialyzed RRL, 10 $\upmu$l complete transport buffer, and 2.5 µl of GQDs solution) and another consisting of 2.5 $\upmu$l GQDs and 22.5 $\upmu$l of complete transport buffer.

### Cell culture

The HeLa cell line utilized in this study is procured from the Cell Repository division of the National Centre for Cell Science (NCCS), India. The cells are cultured in Dulbecco’s Modified Eagle Medium (DMEM) supplemented with 10% Fetal Bovine Serum (FBS), 50 U/ml penicillin, and 0.05 mg/ml streptomycin. The culture is maintained at $37\;^{\circ }$C and 5% CO2. Cells are grown in an imaging chamber (confocal dish) for live cell imaging with approximately 20,000 cells in 100 $\upmu$ l of complete media. The cells are incubated for 12 h before the experiment. The imaging chambers are prepared in-house by drilling a 6 mm hole in a 35 mm petri dish and fixing a coverslip to the bottom.

### Experimental methods

Experiments on the nuclear transport of GQDs are carried out in the time-lapse imaging mode using a confocal fluorescence microscope (FV 3000, Olympus Corporation), equipped with a 60X oil immersion objective. To initiate the experiment, the imaging chamber containing cells is positioned on the microscope stage of the confocal fluorescence microscope. The cells are first observed under bright field illumination. The cells are washed thrice with the transport buffer, to remove any cell debris or unattached cells. Subsequently, cells are treated with digitonin for 5 min to permeabilize the cell membrane. Following permeabilization, the cells are again washed three times with 30 $\upmu$l of complete transport buffer.

The transport buffer is carefully removed from the well, and the import mixture is added without disturbing the position of the imaging chamber. The confocal imaging is initiated in the time-lapse mode of the 405 nm channel just before adding the import mixture. Time-lapse confocal images are acquired for 5 min at a frame rate of 0.625 per s. This captures the fluorescence intensity from the nuclei’s central cross-section as a time function. Once nuclear import saturation is achieved, time-lapse export studies are conducted. The excess import mixture in the imaging chamber is removed completely, and 60 µl of complete transport buffer is added. At this stage, since the concentration of GQDs inside the nucleus is much higher, the GQDs start diffusing out of the nucleus. Time-lapse confocal imaging is performed for 5 minutes to capture this diffusion. Multiple export studies are conducted to verify the retention of GQDs inside the nucleus. After the first export study, the transport buffer solution is removed completely, and a fresh complete transport buffer is added to the imaging chamber. This ensures that the concentration of GQDs outside the nucleus is zero at the beginning of each export study. This export protocol is repeated three times.

To verify that the nucleus is intact during imaging, a control experiment is conducted at the end of each export study, using TRITC-labeled dextran molecules with a molecular weight of 70 kDa, which is much larger than the passive diffusion limit. The experiment involves adding the import mixture containing 70 kDa dextran to the imaging chamber and monitoring the fluorescence inside the nucleus. An intact nucleus will not exhibit any increase in fluorescence over time, and the nucleus remains dark.

Fiji (Image J), an open-source software, is utilized for image analysis. The raw images are analyzed by measuring fluorescence intensity inside the nucleus, excluding the nuclear envelope. Normalized fluorescence intensity is determined by taking the ratio of the fluorescence intensity inside the nucleus to that outside.

## Results

### Nuclear import studies

Time-lapse confocal imaging of GQDs is performed by utilizing their intrinsic autofluorescence property, with an excitation wavelength of 405 nm. Three distinct experiments are carried out. (A) The nuclear entry of GQDs is monitored in HeLa cell nuclei using an import mixture containing RRL, GQDs, and the complete transport buffer. (B) The nuclear entry of GQDs is monitored in HeLa cell nuclei using an import mixture containing only GQDs, and the complete transport buffer. (C) The nuclear entry of GQDs is monitored in HEK 293 cell nuclei using an import mixture containing GQDs and the complete transport buffer. Studies are carried out in these three different systems to understand the influence of RRL and the cell line on the transport process. The time-lapse confocal videos are available in supplementary information (S3).

**Figure 1:**
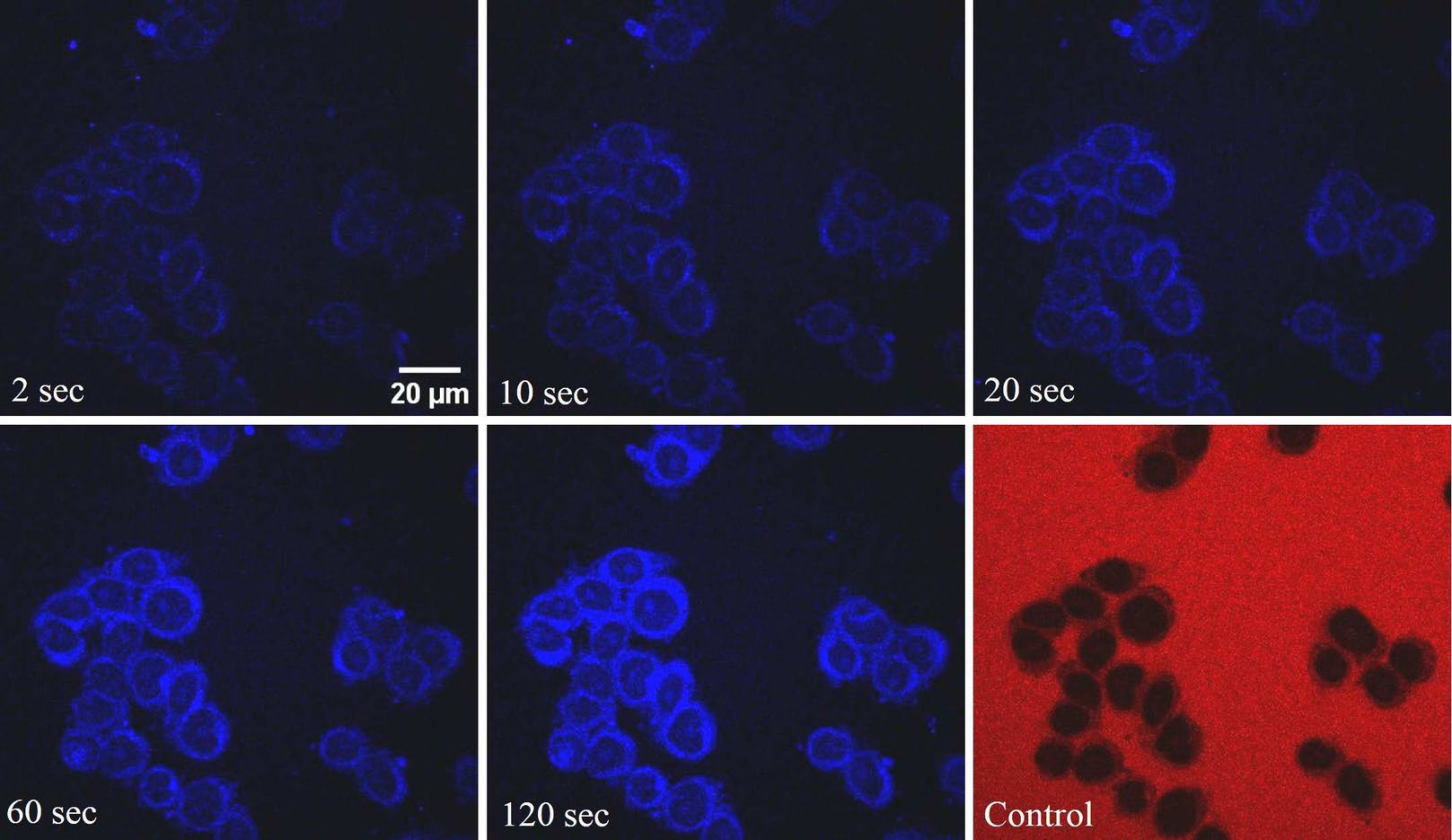
Time-lapse confocal images depicting the nuclear uptake of GQDs in HeLa cell nuclei. The frames show the image of the central cross-section of the nuclei after the addition of the import mixture containing RRL and GQDs, at times indicated. The last frame shows results of a control experiment with 70 kDa TRITC dextran. Scale bar is 20 µm.

Figure 1 presents images of the central cross-section of the nuclei captured at various time points after the addition of the import mixture containing GQDs and RRL (case A). The images depict a rapid entry of GQDs into the nucleus, distributing themselves within the nucleoplasm. It is noteworthy that the diffusion rate within the nucleoplasm is considerably higher than the transport rate through the nuclear membrane. Additionally, it is observed that nuclear organelles such as nucleoli and nuclear membranes exhibit greater brightness compared to other parts of the nucleoplasm, which makes GQD a nucleoli marker (Fig. S2).

**Figure 2:**
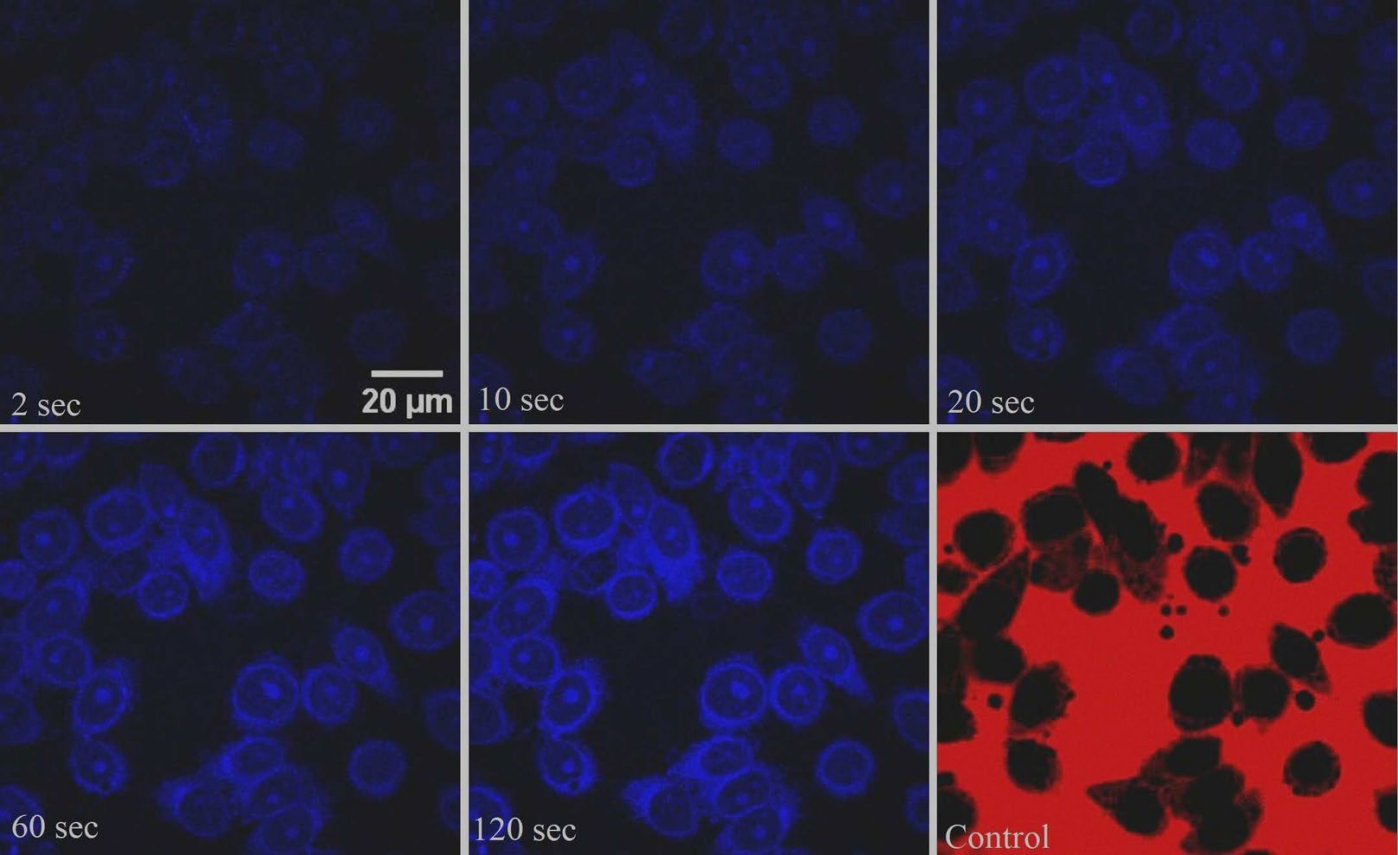
Time-lapse confocal images depicting the nuclear uptake of GQDs in HeLa cell nuclei. The experiment is carried out using an import mixture not containing RRL. The last frame shows results from the control experiment with 70 kDa TRITC dextran. Scale bar is 20 µm.

Figure 2 displays time-lapse images of the nuclear import in case (B), where no RRL was added to the import mixture. The images and the analysis show that the nuclear uptake behavior and the transport rates (Table 1) are similar to those observed in case (A) where RRL is present in the import mixture. This indicates that the RRL does not play a major role in nuclear transport and that the large values of transport rate noticed are not due to any kind of active transport assisted by RRL.

**Table 1:** The rate constant of transport of GQDs in HeLa cell nuclei determined from the nuclear import and export experiments.

HeLa nuclei study	Rate constant $k_{1}$ ($s^{-1}$)	Rate constant $k_{2}$ ($s^{-1}$)
Import studies with RRL	0.31 ± 0.14	0.008 ± 0.003
Import studies without RRL	0.28 ± 0.14	0.008 ± 0.004
Export studies	0.30 ± 0.14	0.010 ± 0.004

The HeLa cells used in our studies are derived from the cervical cancer cell line. Tumors possess a distinct microenvironment marked by hypoxia, inflammation, and angiogenesis, factors that can influence the efficacy of nanoparticle drug delivery. The atypical architecture and enhanced permeability of tumor tissue can lead to increased retention and accumulation of nanoparticles, potentially enhancing the therapeutic effectiveness of cancer treatments^38,39^. In order to check if this property of cancer cells is contributing to the nuclear accumulation, we conducted experiments on normal cells as well.

**Figure 3:**
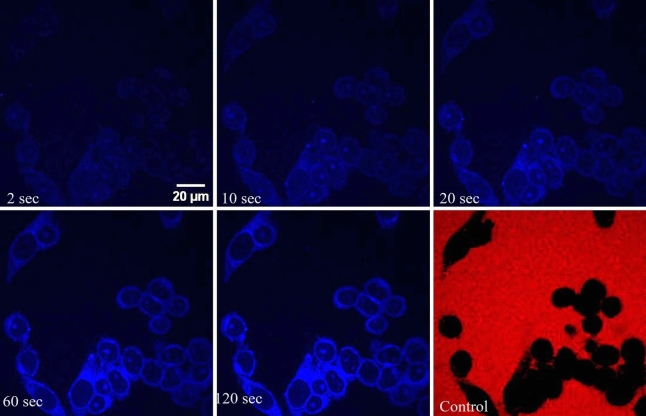
Time-lapse confocal images depicting the nuclear uptake of GQDs in HEK 293 cell nuclei. The last frame shows results from the control experiment with 70 kDa TRITC dextran. Scale bar is 20 µm.

Figure 3 shows that the nuclear uptake behavior of GQDs in permeabilized HEK 293 cell is akin to that observed in HeLa nuclei. The fact that the nuclear transport rates in HEK 293 nuclei are similar to those in HeLa cell nuclei shows that the rapid translocation of GQDs to the nuclei is not due to any specific characteristics of cancer cells.

### Modelling of the transport

The GQDs utilized in the study fall within the passive diffusion limit and are not attached to NLS or importin $\beta$. Hence, it is expected that GQDs are transported to the nucleus via a passive diffusion mechanism, following a first-order kinetic equation represented as:

1 $$\begin{aligned} C(t)/C_0 = A(1-e^{-kt}) \end{aligned}$$

where *C*(*t*) is the concentration of GQDs inside the nucleus, $C_0$ is the concentration outside the nucleus, *k* is the rate constant of transport, and *A* is a constant that has unit magnitude in the case of passive diffusion. This model implies that the transport rate from the cytoplasm to the nucleus is equal to that from the nucleus to the cytoplasm. To analyze the kinetics of transport, the normalized fluorescence intensity inside the nucleus is determined by taking the ratio of the average fluorescence intensity inside the nucleoplasm to the average fluorescence intensity outside the nuclei. As the fluorescence intensity is proportional to the concentration of fluorescing molecules, the normalized fluorescence intensity is equal to the ratio of the concentration, $C(t)/C_0$.

**Figure 4:**
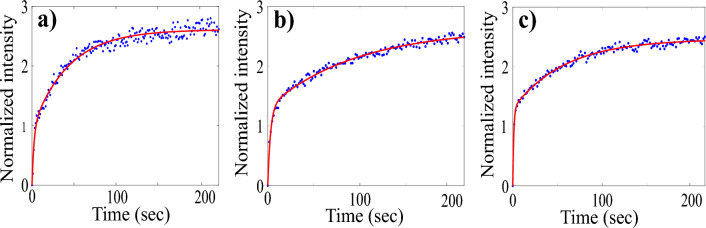
Normalized fluorescence intensity inside the nucleus as a function of time depicting the nuclear entry of GQDs in the case of (**a**) HeLa cell nuclei in the presence of RRL, (**b**) HeLa cell nuclei in the absence of RRL, and (**c**) HEK 293 cell nuclei in the absence RRL. The dots represent experimental data points, and the continuous line is a fit to Eq. (2).

Figure 4 shows the normalized fluorescence intensity inside the nucleus plotted against time. The data deviate from the single exponential behavior described by Eq. (1), suggesting the potential presence of more than one independent transport pathway. If two independent transport pathways exist, the normalized concentration inside the nucleus can be expressed as:

2 $$\begin{aligned} C(t)/C_0 = A(1-e^{-k_1t}) + B(1-e^{-k_2t}) \end{aligned}$$

where A and B are constants. Figure 4 is a plot of the normalized nuclear fluorescence as a function of time. The dots represent experimental data, and the continuous line is a fit to Eq. (2). The good agreement suggests that two distinct mechanisms are involved in the transport of GQDs.

**Figure 5:**
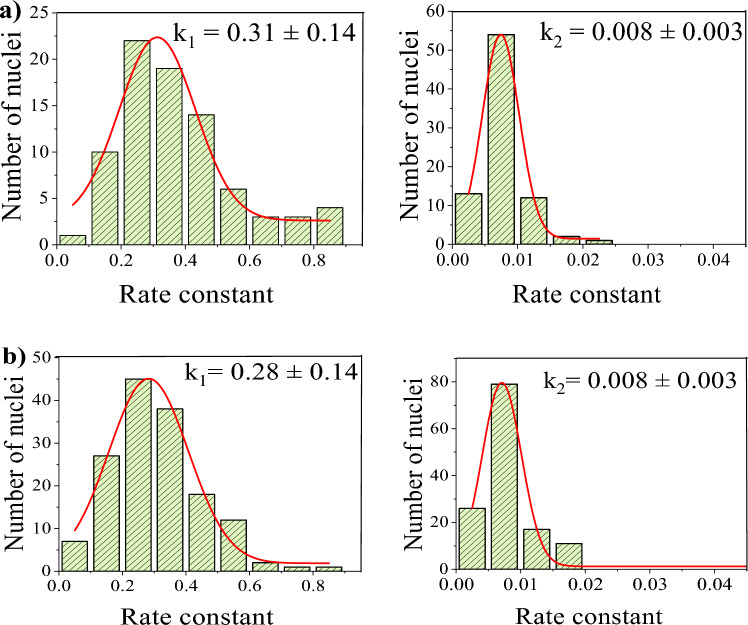
Histogram of the nuclear transport rate constants $k_1$ and $k_2$ in HeLa nuclei (**a**) with RRL and (**b**) without RRL.

The experiment and analysis are replicated on numerous nuclei, and histograms of the translocation rates $k_1$ and $k_2$ of GQDs are drawn to determine the average rate constants (Fig. 5). The analysis reveals two distinct rate constants of transport, $k_1 = 0.31 \pm 0.14$ ($s^{-1}$) and $k_2 = 0.008 \pm 0.003$ ($s^{-1}$) for case A. The transport rates in experiments carried out in case B are also in the same range, $k_1 = 0.28 \pm 0.14$ ($s^{-1}$) and $k_2 = 0.008 \pm 0.004$ ($s^{-1}$). The very similar values of rate constants in both these cases indicate that RRL does not play a significant role in transport. It’s noteworthy that the rate constant $k_2 = 0.008$ ($s^{-1}$) is of the order of the passive diffusion rate constant of dextran molecules of similar size while $k_1$ is much higher^5,7,20^. The import studies in HEK 293 nuclei (case C) also show similar behaviour and rate constants.

### Maximum uptake intensity

In passive diffusion, the concentration inside the nucleus at saturation is expected to equal the outside concentration. However, in our experiments on the nuclear transport of GQDs, we observed that the maximum uptake ratio (ratio of fluorescence intensity inside the nucleus to that outside) is approximately 3. This implies that the concentration of GQDs inside the nucleus is approximately three times higher than that outside. This observation indicates that the GQDs are interacting with nuclear components and getting attached to them. Additionally, the brighter appearance of nucleoli in Figs. 1, 2, and 3 suggests a potential affinity between GQDs and certain nuclear elements.

### Nuclear export studies

**Figure 6:**
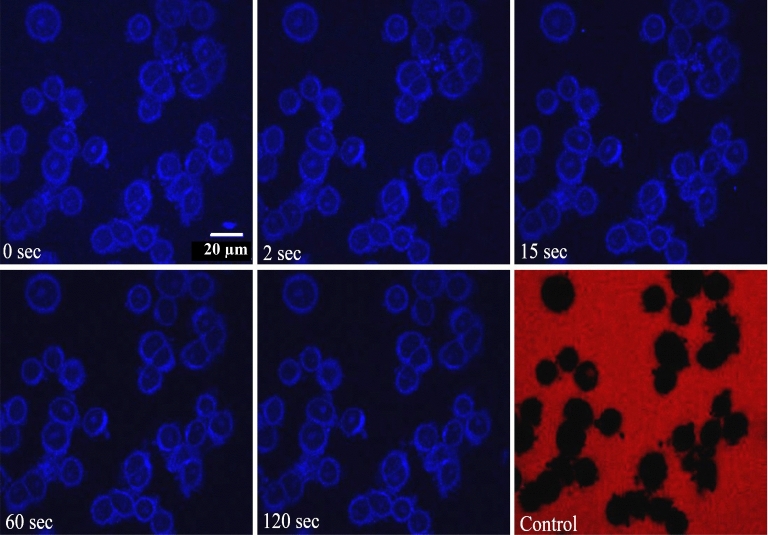
Time-lapse confocal images depicting the export of GQDs from HeLa cell nuclei. The frames show the images of the central cross-section of the nuclei at different times after removing the import mixture. The last frame shows the results of a control experiment with 70 kDa TRITC dextran. Scale bar is 20 µm.

As revealed in the previous section, our data on nuclear import highlights two distinct transport pathways for GQDs’ nuclear uptake. Moreover, the concentration of GQDs inside the nucleus at the transport endpoint is considerably higher than outside. To further explore these findings and understand the bidirectional interaction between GQDs and FG Nups, we directly monitored the export of GQDs from the nucleus. This export study involves removing the import mixture containing GQDs from the imaging chamber after completing the import assay and introducing a fresh, complete transport buffer. Given the high concentration of GQDs inside the nucleus at this stage, the GQDs are expected to diffuse from the nucleus to the surrounding regions due to the concentration gradient. The time-lapse confocal images in Fig. 6 illustrate the diffusion of GQDs from the nucleoplasm. The decreasing fluorescence intensity inside the nucleus with time indicates the export of GQDs from the nucleus. However, it is noticed that the concentration of GQDs inside the nucleus does not go to zero. This suggests that some GQDs are retained in the nuclei after the export assay.

In this scenario, where diffusion occurs from the nucleus (with a high GQDs concentration) to a much larger outside volume (where GQDs concentration approximates zero), the concentration *C*(*t*) inside the nucleus is expected to decrease exponentially,

3 $$\begin{aligned} C(t) = A(e^{-k't}) \end{aligned}$$

where $k'$ is the export rate constant. However, fluorescence intensity inside the nucleus *C*(*t*) does not follow a single exponential curve. Consequently, we attempted to fit the data to a double exponential decay, as described by the following equation:

4 $$\begin{aligned} C(t) = A(e^{-k'_1t}) + B(e^{-k'_2t}) \end{aligned}$$

here, A and B are constants.

**Figure 7:**
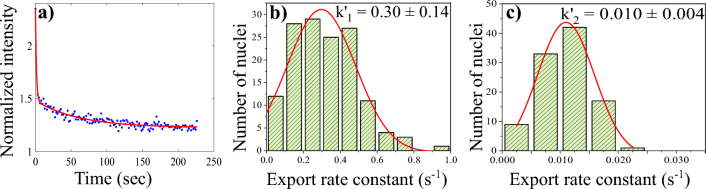
(**a**) Normalized fluorescence intensity inside the nucleus is plotted as a function of time (dots) for nuclear export studies. The solid line is fit to the double exponential decay curve given by Eq. (4). (**b**) histogram of the rate constant $k'_1$ (**c**) histogram of the rate constant $k'_2$.

Figure 7a depicts the nuclear export of GQDs. The solid line is a fit to Eq. (4). The good agreement between the experiment and the model implies two different export pathways. Figure 7b,c depict the histogram of the export rate constants obtained by studying a large number of nuclei. The export rate constants obtained from the analysis are $k'_1 = 0.30 \pm 0.14$ ($s^{-1}$) and $k'_2 = 0.010 \pm 0.004$ ($s^{-1}$). This indicates that the export behaviour of GQDs is similar to that of import, with similar rate constants.

### Multiple export studies and retention

**Figure 8:**
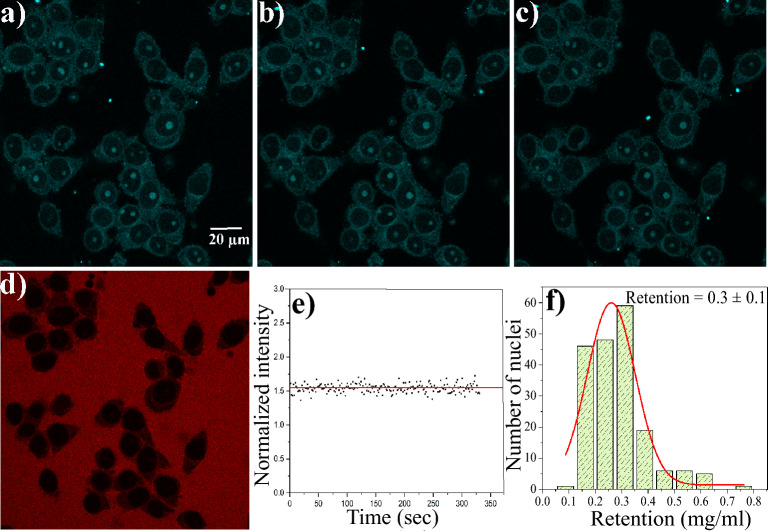
Three successive nuclear export experiments are carried out to study the retention of GQDs in HeLa cell nuclei. Central cross-section of the nuclei at the end of (**a**) first export study, (**b**) second export study, (**c**) third export study, (**d**) control experiment with 70 kDa TRITC dextran. Scale bar is 20 µm, (**e**) graph of normalized nuclear intensity as a function of time during the third export study, (**f**) histogram depicting the concentration of GQDs inside the nucleus at the end of the export studies. The average retention is 0.3 mg/ml.

The findings from export studies indicate that the concentration of GQDs inside the nucleus does not go to zero at the end of the export study. Export experiments are repeated three times to investigate whether GQDs persist in the nucleus. After each study, the nucleus is washed with the complete transport buffer, and the export experiment is repeated. Figure 8a–c depict the final frames of the first, second, and third export studies, respectively, demonstrating consistent fluorescence intensity within the nucleus. Figure 8d shows the results of a control experiment carried out using 70 kDa TRITC dextran, which confirms the integrity of the nuclear membrane at the end of the third export study. Figure 8e shows the normalized nuclear fluorescence intensity during the third export study. The figure shows that the concentration of GQDs remains unaltered even after multiple washes. The experiment was conducted on many nuclei, and the histogram of concentration inside the nucleus is plotted (Fig. 8f). The average concentration of GQDs retained inside the nucleus after multiple washes is 0.3 ± 0.1 mg/ml. The distribution of GQDs is observed across the nucleus, with a more prominent presence in the nucleoli.

## Discussion

The growing potential of nanoparticles as efficient drug-delivery vehicles underscores the need for a comprehensive understanding of their transport mechanisms across cellular and nuclear membranes. Given the limited studies on the kinetics of nuclear transport of nanoparticles, our work focuses on unraveling the transport characteristics of GQDs through the nuclear membrane. This investigation utilizes digitonin-permeabilized HeLa cells and employs time-lapse confocal fluorescence microscopy to shed light on the intricate process of GQD transport.

In contrast to conventional probes like FITC-dextran, GQDs demonstrate unique nuclear transport behaviors, showcasing accelerated translocation and notable retention within the nucleus. Our observations unveil two discernible translocation rates, implying the concurrent operation of two distinct pathways in nuclear transport. The similarity between the rate constant of the slow transport component and that of passively diffusing molecules of similar size suggests that passive diffusion contributes to this component. Meanwhile, the prevalent fast component is likely influenced by mechanisms linked to the surface properties of nanoparticles and their interactions with FG Nups.

The GQDs used in our study lack NLS attached to them, making the possibility of active transport through interaction with importins in the RRL highly unlikely. Consequently, we can posit that, in the conventional sense, active transport does not occur for GQDs. This assertion is also supported by the fact that rate constants remain relatively unaffected by the absence of RRL in the import mixture. The FG Nups in the NPCs, which act as a barrier for transport, carry a positive charge, while all transport factors are negatively charged hydrophobic biomolecules^13,16,18,19^. The charge on both FG Nups and transporting molecules could influence transport rates. Electrostatic interactions between transport factors and FG Nups result in an energy gain, lowering the translocation energy barrier and increasing the transport rate^15,18^. A negative molecular surface charge enhances nuclear pore binding probability and reduces the transport time for passive diffusion through NPCs^17–19^. Thus, we can conclude that electrostatic interactions emerge as a crucial contributor to the efficient translocation of negatively charged GQDs into the nucleus.

The findings from nuclear export studies affirm bidirectional transport, highlighting two export rate constants that closely match their import counterparts. The fast component of the transport, attributed to electrostatic interaction, suggests that the electrostatic interplay between GQDs and FG Nups occurs bidirectionally, implying a signal-less yet interaction-based translocation within the passive limit. The interactive and non-interactive pathways of the bidirectional passive diffusion are identical in both directions.

Multiple export studies corroborate GQD retention in the nucleus, underscoring the significance of surface properties and electrostatic interactions. GQDs trapped inside the nucleus do not actively participate in the diffusion process, resulting in a higher concentration of GQDs within the nucleus at the steady state. The higher concentration of GQDs inside the nucleus than outside, as seen in Fig. 4, can be attributed to the electrostatic interaction between the negatively charged GQDs and the positively charged biomolecules or any hydrophilic linker on the biomolecules present in the nucleus. The GQDs tend to form the $\pi -\pi$ stacking or electrostatic bonding with biomolecules in the nucleus. The nucleus comprises a complex network of biomolecules with varying charges, and these differences in charge create opportunities for the GQDs to bind and remain trapped within the nucleus. The liquid-liquid phase separation within the nucleus may also result in small gaps or voids where the GQDs may be trapped, resulting in a higher concentration^23,31,40^. The fact that the GQDs are retained in the nucleoli region highlights the significance of the surface properties and electrostatic interactions between the GQDs and the biomolecules in nucleoli^31,33,34,40^.

Our study highlights the potential of negatively charged GQDs, as carriers for enhancing the nuclear uptake of biomolecules. The high transport rates into the nucleus and its retention there position GQDs as promising drug carriers for cancer treatment. Further investigation is warranted to unravel the precise mechanisms behind GQD retention, particularly in nucleoli, and to explore other potential biomedical applications.

## Conclusion

In the present work, we use time-lapse confocal fluorescence microscopy to study the kinetics of the transport of negatively charged GQDs through the nuclear membrane. The study reveals a rapid nuclear uptake of GQDs, pointing to the potential application of GQDs as a drug carrier. Results suggest that the presence of RRL does not influence the nuclear uptake of GQDs and that the high nuclear transport rates observed could be due to electrostatic interaction. The nuclear uptake studies in HeLa cells and HEK 293 cells show that the interaction of GQDs with the nucleus is independent of the cell line and is not specific to cancer cells. The export studies confirm the bidirectional nature of transport.

### Supplementary Information

Supplementary Information.

## Data Availability

The data are provided in the manuscript. The raw data used or analyzed in the present study are available from the corresponding author upon reasonable request.
