# Supplementary material for: Role of charge in enhanced nuclear transport and retention of graphene quantum dots
Source: Sci Rep. 2024 Aug 16;14:19044. doi: 10.1038/s41598-024-69809-2 (PMC11329721; doi:10.1038/s41598-024-69809-2)
Supplement: Supplementary file 1 — Supplementary Information. [file 41598_2024_69809_MOESM1_ESM.docx]

**Supporting Information**

**Role of charge in enhanced nuclear transport and retention of graphene quantum dot**

Gorav Gorav, Vrushali Khedekar, Geetha K. Varier, and P. Nandakumar

**GQDs characterization**

GQDs were synthesized from pyrene by a hydrothermal method. The nitration of pyrene was done to get a nitropyrene derivative, which, upon hydrothermal treatment in an alkaline medium, gave adequate hydroxyl functionalities in GQDs [1]. The sample is dialyzed using a dialysis membrane of pore size 2.4 nm to remove the extra chemicals. The characterization tools used are UV-visible spectroscopy, dynamic light scattering (DLS) measurements, zeta potential, transmission electron microscopy (TEM), field emission scanning electron microscopy (FESEM), and Fourier-transform infrared (FTIR) spectroscopy. The UV-visible spectrum, Fig.S1 (a) of GQD, shows a broad peak at 370 nm. We used DLS for particle size determination and observed that the hydrodynamic size of GQDs is 10.4 nm, as shown in Fig. S1 (b). The particle size characterization has also been done using TEM and FESEM imaging, as shown in Fig.S1 (c) and (d). The size of GQDs was found to be approximately 6 nm and 10 nm, respectively in these studies. Zeta potential studies are shown in Fig.S1 (e). The GQDs used in nuclear transport studies are negatively charged with a zeta potential value of -32.4 mV. To find the functional group present on the GQD, we conducted FTIR measurement. As shown in Fig. S1 (f), peak 1 at 3291 (cm^-1^) shows the presence of the OH group of the carboxyl group, and peak 2, 1660 (cm^-1^), shows the presence of C=O.


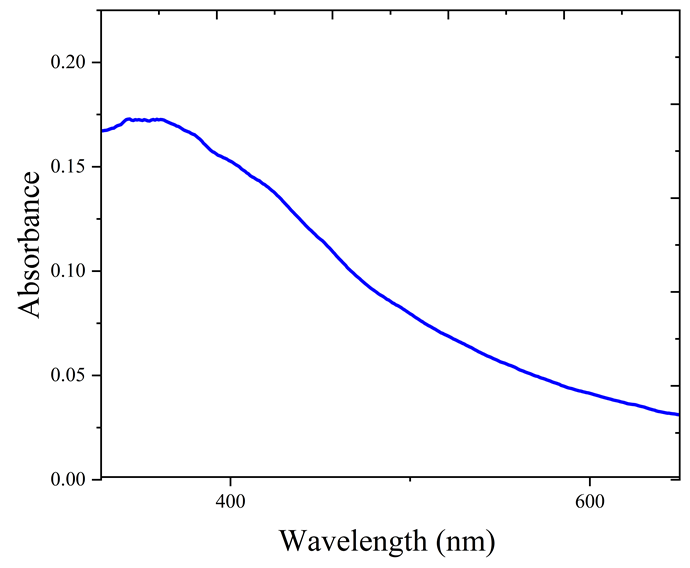


Fig.S1 (a): The UV-Visible spectrum of GQDs shows an absorption peak at 370 nm.


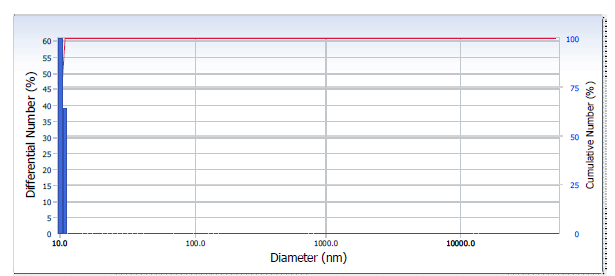


Fig. S1 (b): DLS measurement of GQDs. It can be seen from the graph that the diameter is 10.4 nm with a standard deviation of 0.4 nm.


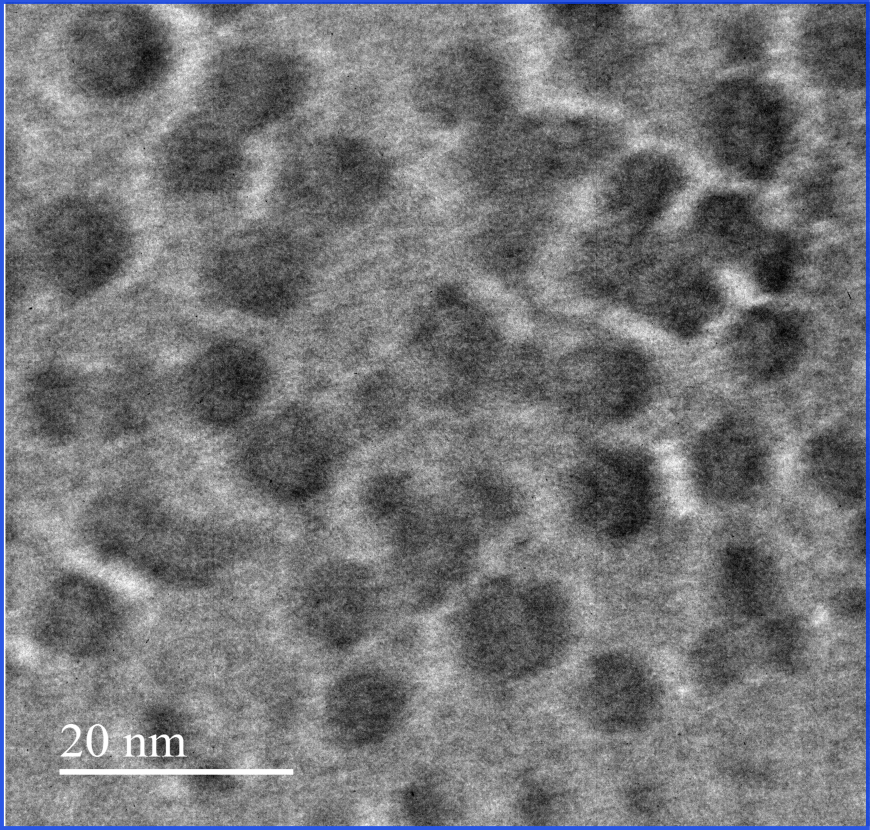


Fig. S1 (c): TEM image of GQDs, the average diameter is 6 nm.


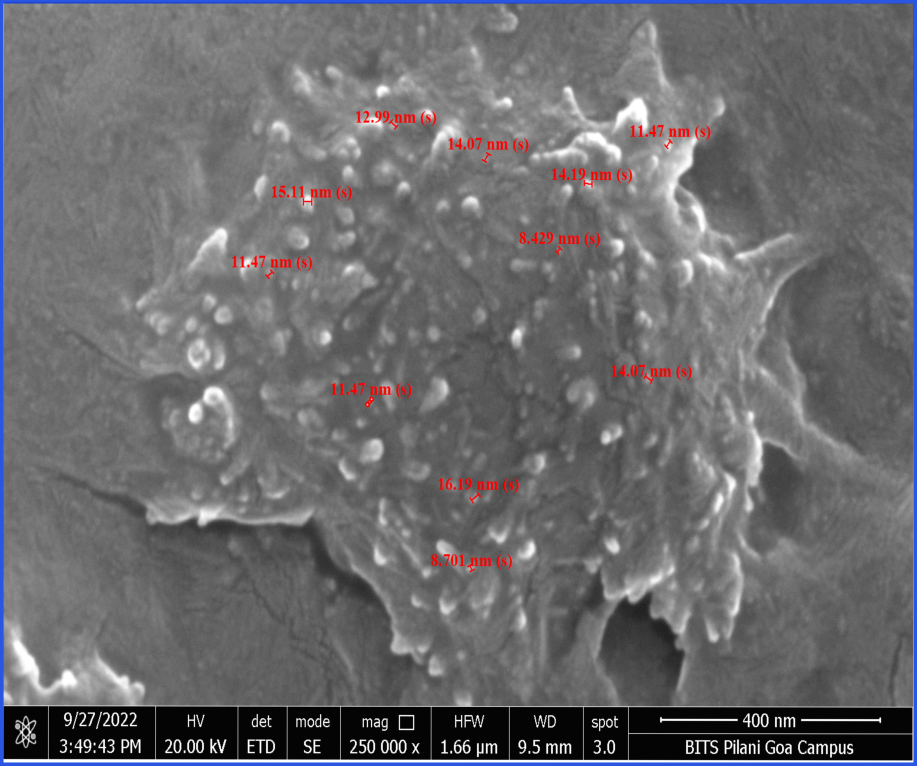


Fig.S1 (d): FESEM image of GQDs, the average diameter is 10 nm.

**
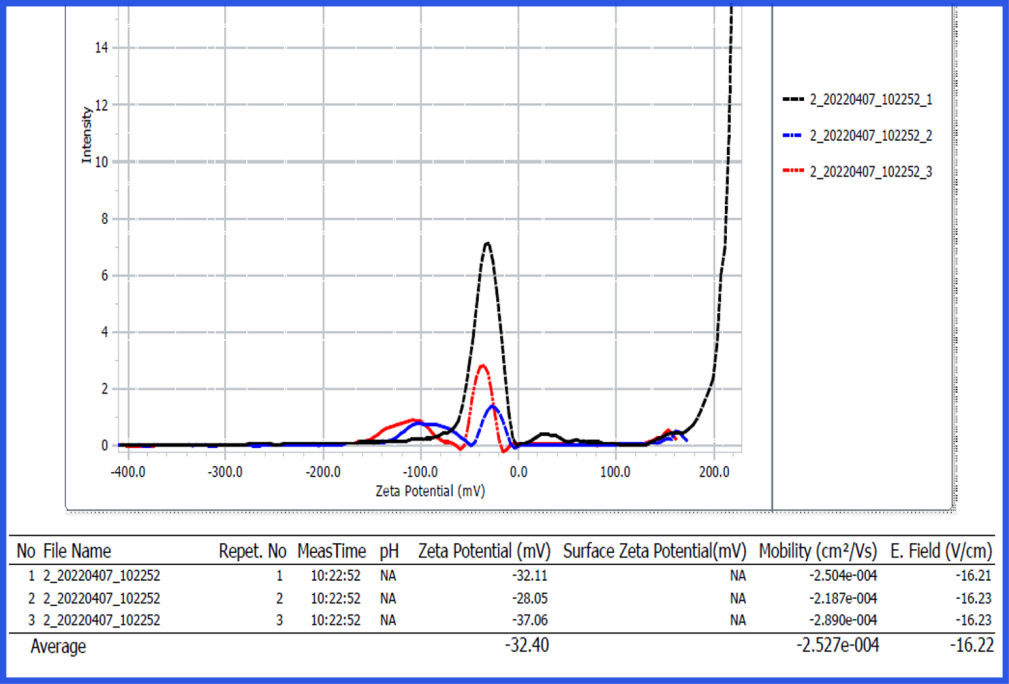
**

Fig.S1 (e): Zeta potential measurement of GQDs for three repeats. The average value is -32.4 mV.


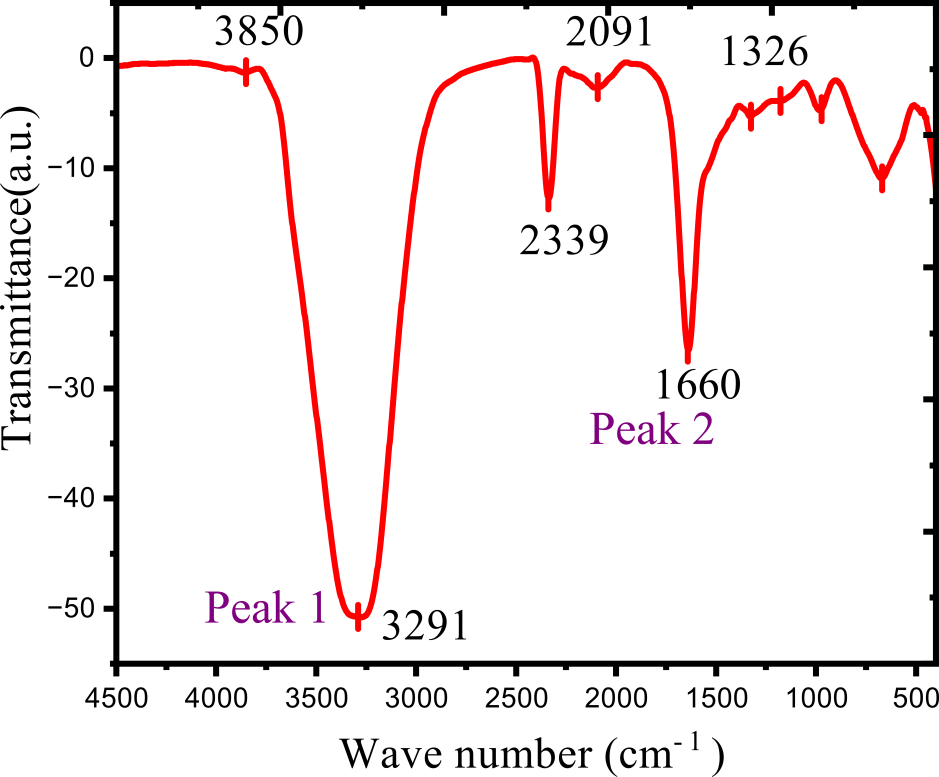


Fig.S1 (f): FTIR curve obtained for GQDs. Peak 1 shows the presence of the OH group of the carboxyl group. The peak 2 shows the presence of C = O.

**Conversion of fluorescence intensity into the concentration of GQDs**

$$Normalized fluorescence intensity \boldsymbol{=}\frac{Fluorescence intensity inside nuclei}{Fluorescence intensity outside nuclei}$$

$$= \frac{Concentration inside nuclei}{Concentration outside nuclei}$$

The concentration of GQDs in the import mixture is 0.2 mg/ml. It is assumed that the concentration outside remains constant since the volume of the imaging chamber is very large compared to that of the nucleus.

GQDs concentration inside nucleus = (0.2 mg/ml)*(Normalized fluorescence intensity)

**GQDs stain the nucleoli and the nuclear membrane**


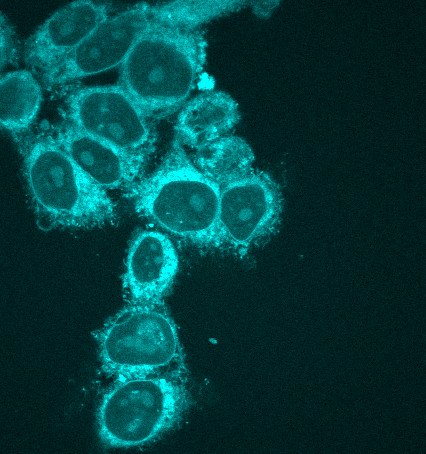

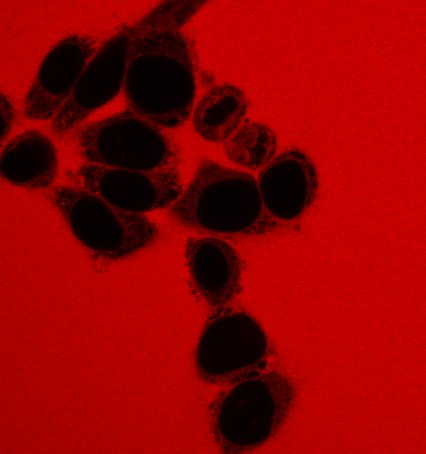


b)

a)

Figure S2. a) The frame shows the image of the central cross-section of the HeLa nuclei after adding the import mixture containing GQDs. It is observed that nuclear organelles, such as nucleoli and nuclear membrane, exhibit greater brightness compared to other parts of the nucleoplasm. b) The frame depicts the results of the control experiment with 70 kDa TRITC dextran.

**Videos of Nuclear Import Studies:**

Video S3: Time-lapse confocal videos of nuclear import of GQDs in HeLa and HEK 293 cells.

**References:**

**[1]** Bhosle, Akhil A., et al. "A combination of a graphene quantum dots–cationic red dye donor-acceptor pair and cucurbit [7] uril as a supramolecular sensor for ultrasensitive detection of cancer biomarkers spermine and spermidine." *Journal of Materials Chemistry B* 10.40 (2022): 8258-8273.
